# Development of a Multimodal Analgesia Protocol for Perioperative Acute Pain Management for Lower Limb Amputation

**DOI:** 10.1155/2018/5237040

**Published:** 2018-06-03

**Authors:** Roberta De Jong, Alexander J. Shysh

**Affiliations:** ^1^Acute Pain Service, Alberta Health Services, Department of Anesthesia, Peter Lougheed Centre, 3500-26 Avenue NE, Calgary, AB, Canada T1Y 6J4; ^2^Cumming School of Medicine, Department of Anesthesia, Peter Lougheed Centre, 3500-26 Avenue NE, Calgary, AB, Canada T1Y 6J4

## Abstract

Multimodal analgesia may include pharmacological components such as regional anesthesia, opioid and nonopioid systemic analgesics, nonsteroidal anti-inflammatories, and a variety of adjuvant agents. Multimodal analgesia has been reported for a variety of surgical procedures but not yet for lower limb amputation in vasculopathic patients. Perioperative pain management in these patients presents a particular challenge considering the multiple sources and pathways for acute and chronic pain that are involved, such as chronic ischemic limb pain, postoperative residual limb pain, coexisting musculoskeletal pain, phantom limb sensations, and chronic phantom limb pain. These pain mechanisms are explored and a proposed protocol for multimodal analgesia is outlined taking into account the common patient comorbidities found in this patient population.

## 1. Introduction

Multimodal analgesia, a concept first articulated by Kehlet and Dahl [1], is now the foundation for the management of acute postoperative pain. Principle pharmacologic elements of multimodal analgesia (Table 1) may include the combination of regional anesthesia (including single-shot or continuous central neuraxial or peripheral nerve blocks and/or local infiltration analgesia), opioid analgesics, and nonopioid systemic analgesics (acetaminophen, and nonsteroidal anti-inflammatories) [2–4]. In addition, pharmacologic adjuvants may be added such as gabapentinoids (e.g., gabapentin and pregabalin), N-methyl D-aspartate (NMDA) receptor antagonists (ketamine, memantidine, dextromethorphan, and magnesium), alpha-2 adrenergic agents (clonidine), glucocorticoids (dexamethasone), and others (antidepressants, calcitonin, nicotine, capsaicin, cannabinoids, and lidocaine) [5, 6]. The premise behind multimodal analgesia is the apparent synergy between agents in interfering with pain pathways at multiple anatomic and pharmacologic sites while limiting side effects overall, specifically through opioid-sparing effects [4, 7].

Patients will benefit from multimodal combinations that hold favourable profiles when considering the specific requirements of both their particular surgical procedure and their medical comorbidities [2, 3, 8]. Lower limb amputation (LLA) in vasculopathic patients presents such a unique challenge, since anticoagulation issues are common in the perioperative period as well as the prevalence of significant cardiopulmonary disease [9]. Furthermore, practitioners must consider not only acute postoperative pain but also chronic ischemic pain, residual limb pain, phantom limb sensations, musculoskeletal pain, and the possible development of phantom limb pain [10]. Postoperative pain for LLA patients is complex and likely involves multiple pain etiologies and pathways [10, 11]. As such, multimodal analgesia may be most valuable as a means by which to treat these multiple and complex pain mechanisms.

Multimodal analgesia has not been described in this patient population as it has been for other surgical procedures [2, 8]. This article summarizes the underlying complexity of pain in LLA patients as well as the development and application of a multimodal analgesia protocol for postoperative pain management. This protocol specifically addresses the unique considerations of the vascular amputation patient relative to their common medical comorbidities together with surgical concerns. Although the primary goal of this review is to mobilize existing literature to enhance analgesia following LLA, it also serves to illustrate how to combine multiple analgesic modalities in a manner that considers the distinctive needs of a specific patient population.

## 2. The Underlying Complexity of Pain in Lower Limb Amputation Patients

Lower limb amputations are commonly performed as a consequence of long-term damage from peripheral vascular disease and diabetes mellitus. The complexity and challenge in managing pain in patients undergoing LLA begins with our limited understanding of the exact pathophysiology and mechanisms underlying the preamputation and postamputation phenomena. These patients may experience several different types of pain (individually or concomitantly) following LLA (Table 2). In fact, LLA is an operative procedure known to have one of the higher incidences of persistent postsurgical pain [18]. Thus, a challenge exists to adequately address these varied pain components.

The majority of patients who require LLA typically have lengthy histories of ischemic limb pain and have been established on analgesic regimes that may include high doses of opioid [9]. Therefore, such patients will benefit from the involvement of an Acute Pain Service team to develop a multimodel analgesic regime and optimize their pain management before and immediately after a LLA [9]. Since pain intensity prior to amputation is a significant predictor of developing chronic limb pain, it must be emphasized that aggressive and early treatment of pain preoperatively and postoperatively may be important to attenuate chronic limb pain [11, 12, 19].

Although common, phantom limb pain (PLP), a neuropathic pain, is difficult to prevent and treat since the exact underlying mechanism for the development of this phenomenon remains unknown [11]. While none have been proven, a variety of theories behind PLP have been proposed and, in fact, multiple mechanisms are likely involved including central, spinal, and peripheral components [10, 11, 13, 15, 16, and 20]. Review articles examining the many pharmacological agents used in the treatment of PLP have not elucidated any consensus guidelines for the optimal management of PLP [10, 11, 13, 15, 20–22]. Nonpharmacological strategies have shown limited efficacy (except for mirror therapy). Stress, anxiety, depression, and other emotional factors have been associated with the persistence and exacerbation of PLP [11, 23]. Unfortunately, poor functional outcomes may occur if prolonged PLP interferes with ongoing rehabilitation and prosthetic fitting [16].

Associated musculoskeletal pains such as back, hip, and knee pain have been identified as issues in the majority of LLA patients postoperatively [10]. Such coexisting pain must not be overlooked, as this may also contribute to significant impairment in function and less favourable rehabilitation outcomes. Optimizing pain management in order to promote mobility and restoration of function is essential since the goal is to start fitting the patient for a tailor-made prosthesis early in their recuperation.

## 3. Development of a Multimodal Analgesia Protocol for Lower Limb Amputation Patients

Lower limb amputations are commonly performed on individuals who are predominantly elderly and have significant comorbidities. In the vasculopathic patient, significant coronary atherosclerosis is common. In fact, LLA may be associated with a 30-day mortality as high as 17% [9]. Prior to prescribing any medications to LLA patients, comorbidities such as cardiovascular disease, cardiac conduction disorders, renal dysfunction, liver disease, and depression must be taken into account since dosing and level of monitoring may need to be adjusted accordingly [24]. Additionally, the potential for adverse effects such as postoperative nausea and vomiting, pruritus, urinary retention, constipation, and respiratory depression are other factors to be considered when managing pain in this primarily elderly population [25]. Perioperative anticoagulation issues also need to be deliberated for the surgical patient with vascular-occlusive disease. All of these factors will impact upon the analgesic plan developed by clinicians to enable tailoring the approach in managing a particular LLA patient's pain.

An assortment of pharmacological agents has been proposed for use in LLA, which may be utilized in a multimodal analgesia regime (Table 3). It is important to reaffirm that although each class of agent is discussed separately in this paper, they work at different sites along the nociceptive pathway and, when combined, will provide more effective analgesia than a single-mode regime [36].

### 3.1. Regional Anesthesia

With respect to multimodal analgesia, perhaps the most profound opioid-sparing effect may be seen with the use of regional anesthesia. Immediately following amputation, there is a continuous barrage of painful sensory input that results in inflammatory changes both peripherally and centrally. Regional anesthesia interferes with the transmission of painful stimuli along the pain pathway to the cerebral cortex [37].

Over the years, a variety of regional anesthesia techniques have been described in the treatment of LLA. Initial reports suggested promise with the use of epidural analgesia preoperatively to prevent or reduce PLP for amputation patients [38]. However, contemporary reviews have reported an inconsistent benefit of preemptive epidural analgesia (or other early regional blocks) before limb amputation to avert PLP despite improved analgesia in the acute pain management phase [10, 39, 40]. There remains a general controversy surrounding the issue of preemptive analgesia and its favourable effects on postoperative pain relief [41].

In contrast, a recent study did show that optimized epidural or systemic analgesia initiated 48 hours preoperatively was indeed effective in reducing PLP at 6 months [19]. In fact, studies that demonstrate effectiveness of preemptive analgesia were those more likely to have initiated the therapy earlier, at least 24 hours or more preoperatively [10]. This again emphasizes the point that aggressive, early treatment of pain may mitigate the severity of postoperative pain for LLA patients. Interest in preemptive analgesia continues, now highlighting the importance of “preventative analgesia,” where adequate and effective attenuation of peripheral and central sensitization to noxious stimuli is provided throughout the preoperative, intraoperative, and postoperative phases [42]. Notably, multimodal analgesia, broadening well into the preoperative phase, is suggested as a means by which to provide such preventative analgesia [43].

Anticoagulation issues, however, are common in the perioperative period for the vascular amputation patient which may preclude the use of central neuraxial regional anesthesia techniques. The perineural analgesia technique obviates such concerns, while providing benefits of an extended regional blockade of painful somatic stimuli postoperatively. Unlike continuous epidural or spinal anesthesia, continuous perineural analgesia is simple to administer, circumvents risks and costs, and avoids the complications of hemodynamic alterations in this highly susceptible patient population [26, 44, 45].

### 3.2. Perineural Analgesia

Fisher and Meller introduced the use of an intraincisional nerve block catheter, positioned at the distal end of the sciatic or posterior tibial nerve, which is inserted by the surgeon at the time of lower limb amputation [26]. Patients receive a general or central neuraxial anesthetic. Intraoperatively, the surgeon dissects the sciatic or posterior tibial nerve during amputation. After the nerve has been transected, a multiport epidural catheter is placed into the wound distally from the main incision and advanced into the nerve sheath above the level of the amputation [46]. The “epidural” (stump) catheter exits the skin through a separate stab incision and is anchored to the skin with the stump dressing. This “epidural” (stump) catheter is now used as a continuous peripheral nerve block (CPNB) catheter. A bolus dose of local anesthetic (e.g., 10–20 mL of 0.25% bupivacaine) is injected into the catheter by the anesthesiologist to confirm placement prior to closure of the wound and to provide regional analgesia. Local anesthetic at a low dosage and rate is then continuously infused through the peripheral nerve block (PNB) catheter (stump catheter) following surgery to provide analgesia and lessen the impact on motor function. A standard patient-controlled epidural analgesia (PCEA) pump is utilized, typically with just a background continuous infusion programmed into the pump. This infusion is continued usually until the first dressing change on postoperative day five, when the “epidural” stump catheter is simply removed.

This surgically placed sciatic CPNB catheter infusing local anesthetic has been shown to reduce opioid requirements and provide improved pain relief immediately and in the early postoperative phase following LLA [27, 28, 47] (one study, however, did not replicate such benefits with this technique [48]). Furthermore, pain relief is achieved with minimal effect on sensory or motor function, which supports early rehabilitation by encouraging movement and reducing edema in the residual limb [46, 47]. Application of this technique should also result in improved respiratory and cognitive function for this elderly vascular population after LLA surgery, thereby accelerating function back to normal and improving monitoring for successful recovery.

Opioids are not added into CPNB infusions but are ordered concurrently, either orally or parentally, with other nonopioid analgesics to manage patients' pain following LLA. Complications related to this technique are rare. Patients in Fisher and Mellor's study did not complain of PLP for approximately one year following amputation [26]. Unfortunately, other investigators using a peripheral nerve sheath catheter have not replicated this protective effect on the development of PLP [20, 22].

The optimal duration for CPNB infusions is unknown, but an average duration of treatment of 4 to 5.5 days following LLA has been described [27, 28, 47, 48]. Of interest is the report that prolonged administration (average 30 days) of various peripheral neural blockades for lower extremity amputations (at a range of anatomic levels) may reduce the incidence to only 3% of severe-to-intolerable PLP at 12 months postoperatively [29]. This potential beneficial impact on chronic PLP needs to be underscored. Katz and Melzack [49] found that 57% of patients with persistent PLP following amputation described it as being similar to the pain they had experienced before surgery, despite the affected limb having been amputated. In view of the extremely high overall incidence of PLP (up to 85%) [11] and its significant negative impact to the patient, extended postoperative CPNB catheter infusions certainly warrant further study.

### 3.3. Opioids

Opioids remain the mainstay for treatment of acute pain following any surgery. Choice of opioid and dosing should be individualized and determined by the patient's preoperative opioid requirements, age, liver, and renal function. Long-term use is not recommended as patients may develop tolerance, chemical dependence, and the potential for opioid-induced hyperalgesia [50]. In fact, one observational study has shown an association between the preamputation use of opioids as a risk factor in the eventual development of PLP [51].

In contrast, there is some evidence that opioids may interrupt central cortical reorganization where PLP is thought to originate [52]. Morphine, given orally or intravenously, has been shown to reduce PLP in the short term but is complicated by notable side effects such as constipation, sedation, tiredness, dizziness, sweating, voiding difficulty, vertigo, itching, and respiratory problems [15, 20, 21].

### 3.4. Systemic Nonopioid Analgesics

Nonopioid and nonsteroidal anti-inflammatory drugs (NSAIDs) are appropriate for alleviating postsurgical inflammatory pain but not for the prevention of neuropathic PLP. Acetaminophen is an effective analgesic for mild to moderate pain, results in few side effects, and has a relatively safe profile. It is recommended that acetaminophen be administered only to a maximum 4000 mg daily (for only 3–5 days duration), less for debilitated patients and used cautiously for individuals with liver impairment [24].

It may be reasonable to add a low-dose NSAID in the short term or for treating breakthrough pain, as long as renal function is adequate and there are no contraindications. However, routine or long-term use of NSAIDs is not recommended because of the increased risk for gastrointestinal and renal toxicity in this patient population [9].

### 3.5. Adjuvants

A variety of drugs that have shown promise in the treatment of neuropathic pain have also been investigated in the treatment of acute and chronic pain after LLA. These include gabapentinoids, NMDA receptor antagonists, antidepressants, lidocaine, calcitonin, clonidine, and Botulinum neurotoxin. Unfortunately, some of these agents have not been tested in controlled studies [15]. Several of the studies completed are regrettably at risk of bias, utilize only small sample sizes, are underpowered, lack control groups, possess short follow-up periods, and yield mixed results [10, 11, 15, 20–22]. Thus, the efficacy of these medications is brought into question for either short- or long-term use for pain relief following LLA. Furthermore, many of these adjuvants have significant adverse side effect profiles.

If an adjuvant was utilized preoperatively, it should be resumed in the postoperative multimodal analgesia regimen. If postoperative pain control becomes an issue, the gradual initiation of an adjuvant may be trialled [53]. The overall goal, however, is eventual analgesic reduction and discontinuation as pain severity abates since the utility of some agents in effectively treating PLP has not been well established [10, 15, 20–22].

Gabapentin has opioid-sparing effects that may be beneficial in limiting the development of opioid tolerance, if opioids are also being prescribed. However, its efficacy in treatment of PLP is inconclusive, with dose-limiting side effects of somnolence, dizziness, headache, and nausea [10, 13, 15, 20–22]. Pregabalin, while showing some success in the treatment of neuropathic pain, also has dose-limiting side effects; no definitive studies examining its efficacy in treating PLP have been reported [15].

The NMDA receptor antagonists ketamine and dextromethorphan have provided some benefit in reducing PLP in the short term [10, 11, 20–22]. However, such use of ketamine has exhibited side effects of loss of consciousness, sedation, hallucinations, hearing and position impairment, and insobriety [21]. Inconclusive results in reducing PLP were found with memantine therapy [15].

With regard to antidepressants, amitryptyline was determined to have inconsistent benefits in the treatment of PLP [10, 13, 15, 21, and 22]. However, one article reported success in abolishing PLP with amitriptyline and tramadol [33]. In addition, there is a case report of four patients who exhibited a marked (>50%) reduction in PLP with the use of mirtazapine [35].

Only variable results were observed in the treatment of PLP with calcitonin [10, 11, 13, 15, and 21]. No benefit was found with the use of capsaicin or Botulinum neurotoxin [11, 21].

## 4. Application of the LLA Perioperative Multimodal Pain Management Protocol

A “best practice” multimodal protocol for managing pain following LLA was created for vascular patients at our acute care hospital (Table 4). This protocol employs the benefit of a CPNB stump catheter for the regional anesthesia component of the multimodal analgesia, which is quick and easy for the surgeon to place during surgery, requiring only an epidural insertion kit and no additional special equipment. It is uncomplicated for staff on patient care units to set up, use, and monitor, even if the patient is anticoagulated. The majority are run as a simple continuous infusion of 0.25% bupivacaine at 5–8 mL/h; rarely is a patient-controlled bolus used. This stump catheter is removed with the first dressing change on postoperative day five. Oral oxycodone is commonly used as a principle opioid; only occasionally is a narcotic PCA pump deemed necessary and rarely are parenteral opioids needed for breakthrough pain. Acetaminophen and NSAIDs are frequently used when renal and hepatic function are adequate. Adjuvants such as gabapentin, pregabalin, and antidepressants are continued when they were utilized preoperatively.

This protocol was developed to promote a consistent way to relieve pain in the preoperative and early postoperative phases for amputation patients, minimize opioid use and its side effects, facilitate recovery, enable earlier physiotherapy, and enhance functional outcomes. In fact, a recent large study with the use of such stump catheters has indeed confirmed its benefits and safety [47]. Of particular emphasis is the growing opinion that multimodal analgesia protocols are of important benefit in perioperative pain management, especially in elderly surgical patients such as those undergoing LLA [54].

## 5. Conclusions

Perioperative pain management of patients undergoing LLA is indeed complex and challenging. The mechanisms of pathophysiology that underlie the postamputation phenomena of pain remain incompletely understood. Despite these issues, it is hoped that implementation of a strategy utilizing a multimodal analgesia protocol will address and enable pain control management at these multiple complex levels and pathways. Pain following LLA may interfere with an individual's functioning, psychological well-being, and may even result in the development of chronic pain. Thus, it is important for all health care practitioners caring for patients undergoing LLA to be aware of the various analgesic options and interventions available in order to implement an aggressive pain management plan that best promotes recovery and rehabilitation. The multimodal analgesic protocol proposed here represents a compilation of commonly used agents that have been shown to be effective for LLA. Further inquiry is required to develop insight into additional approaches [55] that may result in even more effective ways to control pain and alleviate suffering in patients following LLA.

## Figures and Tables

**Table 1:** Multimodal analgesia: pharmacological components.

	Type	Examples
Principle	Regional anesthesia	Central neuraxial or peripheral nerve block
		Single-shot or continuous catheter
		+/− local infiltration analgesia
	Opioid analgesics	Oxycodone, morphine, fentanyl, hydromorphone
	Systemic nonopioid analgesics	Acetaminophen, nonsteroidal anti-inflammatory drugs (NSAIDs)

Adjuvants	Gabapentinoids	Gabapentin, pregabalin
	N-methyl D-aspartate (NMDA) receptor antagonists	Ketamine, memantidine, dextromethorphan, magnesium
	Alpha-2 adrenergic agents	Clonidine
	Glucocorticoids	Dexamethasone
	Others	Antidepressant, calcitonin, nicotine, capsaicin, cannabinoid, lidocaine

**Table 2:** Complexity of pain associated with lower limb amputation.

	Onset & duration	Comments	References
Ischemic limb pain	Preoperative to intraoperative.	Pain intensity prior to amputation is a significant predictor of developing chronic limb pain.	[11, 12]

Residual limb pain (stump pain)	Intraoperative to 1-2 weeks postoperative. Median pain 15.5 on a 0–100 visual analog scale in the first week postoperatively. Severe pain in 5–10% of patients.	Stump pain (sharp, localized pain) gradually lessens as the wound heals. May be prolonged if complications arise such as infection, tissue necrosis, wound dehiscence, osteomyelitis, and neuroma formation.	[10, 11, 13, 14]

Phantom limb pain	Onset 1–7 days postoperative (or longer). Incidence up to 85%. Mean pain 22 on a 0–100 visual analog scale at 6 months after amputation. Severe pain in 5–10% of patients. May persist for months to years.	Symptoms: Intermittent (or sometimes constant) aching, cramping, burning, shooting, stabbing, boring, squeezing, or throbbing pains. Multiple poorly understood etiologies.	[10, 11, 13, 15, 16]

Phantom limb sensations	Onset 1–7 days postoperative. Incidence up to 90%. May persist for months to years.	Symptoms: Nonpainful sensations that the amputated limb still exists but may feel twisted deformed or have muscle cramps, tingling or itching. Multiple poorly understood etiologies.	[10, 11, 13, 15]

Other musculoskeletal pain	Postoperative.	Back, hip, and knee pain with gait abnormalities related to changes in mechanics due to the amputated limb/prosthetic.	[10, 17]

**Table 3:** Pharmacologic components of multimodal analgesia for perioperative pain relief for lower limb amputation (LLA).

Component	Representative drug/dose	Comments	References
Regional anesthesia		Various regional anesthesia techniques: epidural, spinal, and peripheral nerve block catheters.	
Example: stump catheter	Bupivacaine 0.125–0.25%	Continuous infusion of 4–14 mL/hr (may add bolus 2–5 mL with lockout of 20–60 minutes). Continue for 4–5 days postoperatively.	[26–28]
	Ropivacaine 0.1–0.5%	No benefit in prevention of PLP (of possible benefit if extended over a longer period of time perioperatively).	[19, 29]

Opioid analgesics	Variety of agents	May be of use for short term or breakthrough pain control postoperatively. Use lowest doses that provide adequate analgesia with tolerable side effects. Wean as soon as possible and try to avoid long-term use. Intravenous or oral morphine may reduce PLP but has significant side effects.	[15, 20, 21]

Systemic nonopioid analgesics	Acetaminophen: up to 4000 mg/day for 3–5 days duration	Reduction of dose in debilitated patients. Good safety profile but use cautiously in patients with hepatic impairment.	[13, 24]
	NSAIDS: variety of agents	For breakthrough pain if renal function adequate and no contraindications. Routine or long-term use in the elderly is not recommended due to GI and renal toxicity.	[9, 24, 25]

Gabapentinoids	Gabapentin: 100 mg BID to TID, up to 1200 mg TID maintenance	Start with low dose and gradually titrate to an increased dose every few days, up to 2400–3600 mg/day total dose. Use lower dose if poor renal function. May take several weeks to see peak effect. Dose-limiting side effects of somnolence, dizziness, headache, and nausea. Efficacy for PLP inconclusive, whether started early or late.	[10, 15, 20–22, 24, 30]
	Pregabalin: 50 mg once daily, up to 150 mg BID	Start with single daily low dose and gradually increase to twice daily only after one week, up to 150 mg BID. Consider monitoring renal function. Dose-limiting side effects of drowsiness, dizziness, ataxia, and blurred vision. Efficacy for PLP unknown.	[30]

NMDA antagonists	Ketamine low-dose IV infusion: 0.1–0.2 mg/kg/hr for 24–72 hours (for acute pain) or 0.4–0.5 mg/kg infusion over 45–60 minutes (therapy for chronic PLP)	Caution with hepatic impairment. Contraindicated with elevated intracranial or intraocular pressure, globe injuries, high-risk coronary or vascular disease, history of psychosis, sympathomimetic syndrome, recent liver transplantation, and porphyria. Only limited studies when infusions used for acute pain treatment for LLA. Some reports of short therapeutic infusions for established chronic PLP.	[10, 11, 20, 21, 22, 31]
	Oral dextromethorphan 60–90 mg BID for 10 days (therapy for chronic PLP)	Limited small studies in (cancer) amputees. Dose-related side effects of tachycardia, respiratory depression, nausea, vomiting, hallucinations, and acute changes in memory and cognition. Thus, avoid doses above 2 mg/kg.	[10, 20, 21, 22, 32]
		Ketamine and dextromethorphan (but not memantidine) have shown some benefit in treatment of PLP but are limited by side effects.	[10, 11, 15, 20–22]

Antidepressants	Amitriptyline 25 mg TID (or 50–100 mg once daily at bedtime) titrated to maximum 150 mg/day	For geriatrics, start amitriptyline at 10 mg once daily at bedtime, increase weekly by 10 mg/day. Side effects are dry mouth, drowsiness, sedation, orthostatic hypotension, constipation, urinary retention, weight gain, and arrhythmia. Contraindicated in glaucoma, prostatism, and significant cardiovascular disease.	[10, 11, 21, 22, 33, 34]
	Nortriptyline 25 mg TID titrated to maximum 150 mg/day	For geriatrics, start nortriptyline at 10 mg once daily, increase weekly by 10 mg/day. Similar precautions as per amitriptyline. If adequate pain relief is obtained with amitriptyline but unable to tolerate side effects, consider a trial of nortriptyline.	[13, 30, 34]
	Mirtazapine 15 mg once daily at bedtime titrated to maximum 45 mg/day	For geriatrics, start mirtazapine at 7.5 mg once daily at bedtime.	[35]
		One study showed success in abolishing PLP with amitriptyline and tramadol in young, posttraumatic amputees. One case report of four patients who exhibited a marked (>50%) reduction in PLP with the use of mirtazapine.	[33, 35]

**Table 4:** Perioperative multimodal acute pain management protocol for lower limb amputation (LLA).

Phase	Focus	Multimodal pain management	Comments	Precautions/references
Preoperative	Assess and treat acute or chronic pain before surgery. Optimize an analgesic regime based on the patient's condition.	**Principle components**: Oral +/− parenteral opioids intravenous piggyback (IVPB), or patient-controlled analgesia (PCA), acetaminophen, NSAIDs. **Adjuvants**: gabapentinoids, antidepressants	Consult the acute pain service (APS) if the patient has: (i) Chronic pain issues requiring high dose opioid (ii) A complex pain management regime (iii) Standard analgesics ordered that are ineffective	Aggressive and early treatment of pain is needed to mitigate the severity of chronic limb pain [9, 12, 19, 42, 43]

Intraoperative	Perineural (CPNB) “stump” catheter surgically placed: (i) Below the knee amputation (posterior tibial nerve) (ii) above the knee amputation (sciatic nerve)	**Principle components:** (1) Regional anesthesia CPNB catheter: (i) Bolus with local anesthetic (bupivacaine 0.25%, 10–20 mL) before wound closure, volume dependent on patient size (2) Opioids: intraoperative opioids as needed	For CPNB infusions: (i) Use only nonepinephrine solutions of local anesthetic: (bupivacaine 0.125–0.25%, or ropivacaine 0.1–0.5%)	APS is contacted for all issues related to the CPNB catheter (i.e., disconnection, choice to continue or remove the CPNB catheter) [26, 28]

Postoperative day 0-1	Control residual limb pain (RLP). Maintain the preexisting analgesic regime. Prevent opioid withdrawal.	**Principle components**: (1) Regional anesthesia CPNB infusion: Drug & concentration: (i) Bupivacaine 2.5 mg/mL (2.5% in 250 mL normal saline) or (ii) Ropivacaine 2 mg/mL (2.0% in 100 mL normal saline, recommended with renal dysfunction) Dosing parameters: (i) Continuous infusion 4–10 mL/hr (ii) Start at 5–6 mL/hr, increase by 2 mL/hr at a time every 2 hours (up to 10 mL/hr) if pain persists (2) Opioids: (i) Oral +/− parenteral opioids (IVPB or PCA) for breakthrough pain (ii) Oral opioids (short acting) for opioid-naive patients: oxycodone, morphine, hydromorphone (iii) Commonly used: oxycodone 5–10 mg p.o. q3-4 h PRN or oxycodone 2.5–5 mg p.o. q3-4 h PRN (frail older adult, renal impairment) (3) Nonopioid analgesics: (i) Acetaminophen 500–1000 mg p.o. q6 h (ii) NSAIDs: Ibuprofen 200–400 mg p.o. q6 h or Ketorolac 10 mg IVPB q6 h or Celebrex 100 mg p.o. BID **Adjuvants:** (i) Gabapentinoids: gabapentin or pregablin (ii) Antidepressants	APS follows patients with CPNB infusions and orders all analgesics, antipruritics, antiemetics, and sedating medications. Usually no patient-controlled bolus dose is needed. But if ordered, suggest: (i) Bolus dose 2–4 mL (ii) Lockout 20–30 minutes. Adjust opioid doses for patients already on substantial doses of opioids preoperatively. (i) Continue long-acting opioids for opioid-tolerant patients. (ii) Use parenteral opioids for severe pain not controlled by CPNB infusion and oral analgesics. (iii) Consider PCA if this was used preoperatively or if systemic opioids are required for more than a few hours postoperatively. Acetaminophen ordered around-the-clock for 2–5 days with dosage adjustments after 2-3 days. Add NSAID only if renal function is adequate and there are no other contraindications (e.g., active or previous ulcer disease, systemic anticoagulation, asthma, and congestive heart failure). Resume adjuvants if already established on these agents preoperatively.	Notify APS if the patient complains of persistent pain (>5/10) not relieved with an increase in CPNB infusion rate or other analgesics ordered. CPNB catheter may require a repeat bolus of local anesthetic (as intraoperatively) [27, 28, 46, 47] Protect the affected limb for the duration of CPNB infusion to prevent injury or pressure sores as “numbness” is common (e.g., avoid placing laptop computers, cold or hot packs on areas of skin with decreased sensitivity). Choice of opioid and dosing determined by patient's preoperative opioid requirements, age, hepatic, and renal function [51] Start opioids at low dose and gradually increase to provide adequate pain relief with tolerable side effects [24, 25, 39, 50, and 53] Risk of acetaminophen for hepatotoxicity: maximum daily dose 4000 mg, less for debilitated patients [24, 25] Long-term use of NSAIDs is not recommended due to GI and renal toxicity. Celebrex is a possible alternative to traditional NSAIDs [3, 9, 25] Reduce dose of gabapentinoids with renal dysfunction or if side effects ensue (sedation, drowsiness) [10, 30, 53]

Postoperative day 2–3	Assess and aggressively treat residual limb pain (RLP) and phantom limb pain (PLP).	**Principle components**: (1) Regional anesthesia CPNB catheter: (i) Continue infusion (2) Opioids: discontinue PCA (i) Unless pain intensity remains severe (>5/10) (ii) Transition to oral opioid with IVPB opioid rescue dose PRN (3) Nonopioid analgesics: (i) Acetaminophen: reduce dose to 500–650 mg q6 h (ii) NSAIDs: continue PRN **Adjuvants:** (1) Consider initiating: (i) Gabapentin 100–300 mg p.o. BID to TID, increasing dose every 2–3 days to a maximum of 2400–3600 mg/day, if needed or (ii) Pregabalin 50–75 mg p.o. daily, increasing dose to BID after one week, to a maximum dose of 150 mg BID if needed (2) Continue antidepressants	Adjust CPNB infusion rate and opioid doses along with adjuvant agents to provide adequate pain relief. Initiate gabapentinoid gradually for patients with significant RLP and PLP issues not relieved by CPNB infusion and the “usual” analgesic regime. This may be effective in reducing opioid requirements.	Only use parenteral opioids (IVPB or PCA) in the early postoperative phase to manage pain. Switch to oral opioid as soon as possible [25] Use nonopioid analgesics cautiously in older or frail patients (>65 years), OSA, and renal dysfunction. Lower initial doses with slow titration to manage side effects of dizziness, sedation, and tolerability. Monitor renal function [54] If considering initiating a trial of a new adjuvant, do so only gradually [53] [11, 21, 24, 30, 34]

Postoperative day 3-4	Pain management coordinated with increased activity	Maintain CPNB infusion and multimodal analgesia.	Consider consulting the chronic pain service for optimizing a long-term pain management plan.	Initiate early plans for the eventual analgesic regime, especially for complex pain patients [42, 43]

Postoperative day 5	Discontinue CPNB catheter	CPNB catheter removed with initial dressing change to the residual limb (unless ordered for a longer duration by the anesthesiologist or if requested by the vascular surgery team)	Maximum period of time for CPNB catheter to remain in place: 7 days [27, 28, 47, 48]	Gradually wean patient off opioid and nonopioid analgesics. Adjust adjuvant doses with the overall goal of reduction and/or eventual discontinuation

Postoperative day 6 and following	Management of persistent pain and/or phantom limb pain (PLP), if present	Continue multimodal analgesia agents (not including the CPNB catheter) at lowest possible doses.	Treat persistent pain like neuropathic pain. [18, 30, 34, 53] Reconsider consulting the chronic pain service.	Currently there are no consensus guidelines for the optimal management of chronic PLP [10, 11, 13, 15, 16, 20–22, 39]

IVPB, intravenous piggyback; PCA, patient-controlled analgesia; APS, acute pain service; CPNB, continuous peripheral nerve block; RLP, residual limb pain; NSAID, nonsteroidal anti-inflammatory drug; GI, gastrointestinal; PLP, phantom limb pain; OSA, obstructive sleep apnea.
